# Malaria prevention reduces in-hospital mortality among severely ill tuberculosis patients: a three-step intervention in Bissau, Guinea-Bissau

**DOI:** 10.1186/1471-2334-11-57

**Published:** 2011-03-02

**Authors:** Raffaella Colombatti, Martina Penazzato, Federica Bassani, Cesaltina Silva Vieira, Antonia Araujo Lourenço, Fina Vieira, Simone Teso, Carlo Giaquinto, Fabio Riccardi

**Affiliations:** 1 Clinic of Pediatric Hematology-Oncology, Department of Pediatrics, University of Padova, Padova, Italy; 2 National Reference Hospital for Tuberculosis and Lung Disease, Bissau, Guinea- Bissau; 3 Infectious Disease Unit, Department of Pediatrics, University of Padova, Padova, Italy; 4 University of Padova, Padova, Italy; 5 Department of Public Health and Cell Biology "Tor Vergata" University, Rome, Italy

## Abstract

**Background:**

Malaria and Tuberculosis (TB) are important causes of morbidity and mortality in Africa. Malaria prevention reduces mortality among HIV patients, pregnant women and children, but its role in TB patients is not clear. In the TB National Reference Center in Guinea-Bissau, admitted patients are in severe clinical conditions and mortality during the rainy season is high. We performed a three-step malaria prevention program to reduce mortality in TB patients during the rainy season.

**Methods:**

Since 2005 Permethrin treated bed nets were given to every patient. Since 2006 environmental prevention with permethrin derivates was performed both indoor and outdoor during the rainy season. In 2007 cotrimoxazole prophylaxis was added during the rainy season. Care was without charge; health education on malaria prevention was performed weekly. Primary outcomes were death, discharge, drop-out.

**Results:**

427, 346, 549 patients were admitted in 2005, 2006, 2007, respectively. Mortality dropped from 26.46% in 2005 to 18.76% in 2007 (*p-value *0.003), due to the significant reduction in rainy season mortality (death/discharge ratio: 0.79, 0.55 and 0.26 in 2005, 2006 and 2007 respectively; *p-value *0.001) while dry season mortality remained constant (0.39, 0.37 and 0.32; *p-value *0.647). Costs of malaria prevention were limited: 2€/person. No drop-outs were observed. Health education attendance was 96-99%.

**Conclusions:**

Malaria prevention in African tertiary care hospitals seems feasible with limited costs. Vector control, personal protection and cotrimoxazole prophylaxis seem to reduce mortality in severely ill TB patients. Prospective randomized trials are needed to confirm our findings in similar settings.

**Trial registration number:**

Current Controlled Trials: ISRCTN83944306

## Background

Malaria and Tuberculosis are important causes of morbidity and mortality in Africa [1] and are among the main reasons of hospital admission and in-hospital mortality [2-5]. During the rainy season, malaria burden increases [6-8] in many African countries, peaking morbidity and mortality. Regardless of HIV status, malaria infection affects severely ill TB patients who are already compromised by malnutrition, deprived immunity or disseminated disease [6,9,10]. In fact, in pulmonary TB there is a transient systemic immunosuppression due to over expression of transforming growth factor beta and interleukin-10 [11]. Interactions between TB and malaria have been demonstrated both in vitro and in vivo: Plasmodium Falciparum modulates Mycobacterium Tuberculosis infection [12] and malaria has been shown to exacerbate mycobacterial infection [13]. The reasons for this are not completely explored but seem to involve parasite-parasite interaction and host-parasite interaction [12-14]: malaria causes a further depression in immunity through a qualitative and quantitative defect in T lymphocytes, mainly the CD8+ that are necessary for anti-mycobacterial response, and through a deregulation of the cytokine cascade. Moreover, the respiratory distress that is frequent during acute malaria both in children (due to metabolic acidosis) and adults (due to pulmonary edema and Acute Respiratory Distress Syndrome) [15], can worsen the respiratory effort related to TB. Therefore, given the multiple interactions between malaria and TB and the mutual effect in increasing mortality [1,5,6], new strategies of integrated management should be investigated, especially during malaria peak transmission seasons. The identification of efficacious and low cost methods for reducing malaria in TB patients could largely improve clinical outcome and public health strategies optimizing the use of current limited resources.

Malaria prevention can be performed at various levels and several measures are proven to be quite effective in different groups of patients [16-20].

TB Reference Hospitals can be the sites to test integrated models of malaria prevention for severely ill patients due to the fact that those who are in poor conditions are generally admitted for at least the intensive phase of TB treatment (two months).

In the Hospital Raoul Follereau (HRF), National Reference Hospital for Tuberculosis and Lung Disease in Bissau, Guinea-Bissau, mortality during 2004 rainy season reached peaks of 60% in admitted patients. According to the clinical reports made by the hospital's physicians, high fever with Plasmodium Falciparum positive blood film was the main reason of death. In order to reduce mortality during the rainy season in admitted TB patients, since January 2005 we performed a malaria prevention program in the hospital. Our hypothesis was that the successive combination of personal protection, vector control and cotrimoxazole prophylaxis could be progressively more effective in reducing mortality in severely affected TB patients, who are admitted for long periods of time. Several other drugs with a potential better efficacy are used for malaria prophylaxis, but in the peculiar context of TB Units where almost half of the patients is HIV+ [1,21,22], the use of cotrimoxazole would potentially integrate the interventions and facilitate the long-term administration by the health care workers.

## Methods

### Setting

Guinea-Bissau is located in the West African Atlantic Coast. There are two climatic seasons: a rainy season from July to October and a dry season from November to June. Malaria is endemic with a peak transmission during the rainy season. The Hospital Raoul Follereau (HRF) is the National Reference Hospital for Tuberculosis and Lung Disease in Bissau, capital of Guinea-Bissau. According to the National Guidelines for TB, TB patients in poor clinical conditions or with severe disease (i.e important wasting or respiratory distress) are admitted after referral from regional hospitals or from TB health centers across the entire country.

**Figure 1:**
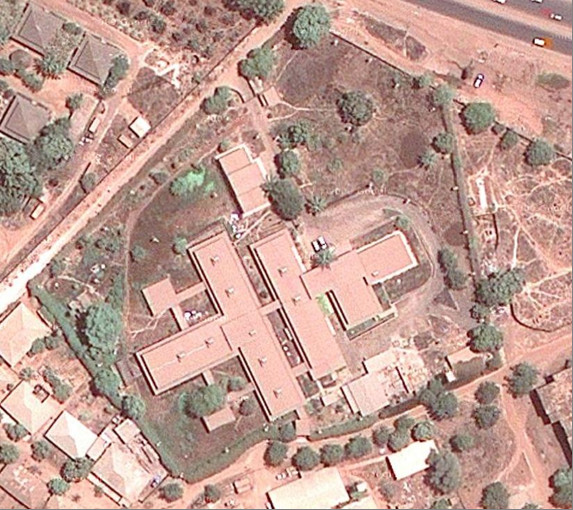
Hospital Raoul Follereau from the satellite http://www.googlemaps.com

After being destroyed during the 1998-1999 civil war, the hospital was rebuilt and reopened to the public in 2004. The hospital is a 2500 sqm structure surrounded by a 7500 sqm grass garden (Figure 1). The whole facility includes an in-patient service (three wards: men, women and children), an outpatient service, a laboratory, two X-Ray Units, a pharmacy, a service area (kitchen, laundry, ironing) and two cafeterias. From 2003 to 2008 the hospital policy was to perform clinical evaluation, diagnostic tests, treatment and nutritional support without charge to all admitted patients.

### Study outline and Malaria Prevention

Malaria prevention was performed through a three-step intervention. A Permethrin treated bed net was given to every patient upon admission since 2005 and during all the three years of the intervention; bed nets were retreated every 30-45 days.

Environmental disinfection was performed monthly with Permethrin based products by appropriately trained hospital staff according to manufactory's instructions during the rainy season of 2006 and 2007; spraying was conducted both outdoor (with Microsin) and indoor (with Petrin L) on doors and window frames. The only difference was that in 2006 environmental outdoor and indoor disinfection was repeated monthly from July 15th to October 30th while in 2007, due to increased availability of the products, it was repeated monthly from June 1st to October 30th.

Prophylaxis with once daily Trimethoprim 80 mg/Sulfamethoxazole 400 mg tablet was given to every patient admitted from July 1st to October 30th only in 2007.

The study was approved by the Hospital Board. All patients gave informed oral consent and all patients benefited from the interventions.

### Diagnosis of TB and Malaria and standard treatment

As part of routine clinical evaluation, patients who were admitted with a suspect of TB, received a three sample sputum analysis, a thorax X-Ray, a complete blood count, a biochemistry analysis and a malaria thick film. Ziehl-Neelsen's sputum staining technique was used and patients were considered smear positive if acid fast bacilli were shown on at least two samples. Smear negative patients were considered to have TB according to the physician's evaluation of chest X-Ray and clinical condition. Additional analyses were performed if necessary, based on physician's judgment.

HIV testing and Antiretroviral treatment were offered free of charge to patients admitted from July to August, in November and in March of every year; alternatively, due to the limited availability of free reagents and drugs, patients were advised to undergo HIV testing in other health facilities and return back the result.

A clinical episode of malaria was defined as an axillary temperature ≥37.5°C together with the presence of malaria parasitemia at any density.

The hospital treatment protocols for TB, Malaria, HIV/AIDS and other diseases were the same in 2005, 2006, 2007. TB was treated according to the National Guidelines, with a four drug regimen for two months (rifampicin, isoniazid, ethambutol and pyrazinamide) followed by two drugs for the following six months (isoniazid and ethambutol); five drugs were used in case of relapsed TB (streptomycin, rifampicin, isoniazid, ethambutol and pyrazinamide). Chloroquine was administered as standard treatment in case of uncomplicated malaria, while severe malaria was treated with iv Quinine, according to the National Program against Malaria in Guinea-Bissau. Other medications were given on a clinical basis. Nutritional support was similarly implemented over the three years and included three main meals and two snacks given daily to all patients.

Compliance to treatment (TB drugs, cotrimoxazole and other drugs) was assured by direct observation of drug intake by the nurses.

Health education on TB, malaria, HIV, water-borne diseases and personal hygiene was part of the hospital care standards: every week an appropriately trained nurse performed health education lessons to in-patients and the number of participants was recorded. The importance of malaria prevention and bed net use was particularly stressed during the lessons.

### Outcomes

Primary outcomes were death or discharge from hospital. Secondary outcomes were drop-outs (defined as voluntary discharge from hospital before recovery against physician's advice and/or treatment abandonment) and percentage of attendance of health education classes.

### Data collection

Personal and clinical information were transferred from patient's individual charts to Microsoft Access 2000 (Microsoft Corporation, Seattle, WA, USA). Hospital registries were used to cross-check diagnosis and admission-discharge dates. Pearson's chi-square test was used to compare variables within groups. *p-values*< 0.05 were considered statistically significant.

## Results

A total of 427, 346 and 549 patients were admitted in 2005, 2006 and 2007, respectively. Major baseline characteristics -gender, symptoms on admission and diagnosis- were similar in the three groups of patients as shown in Table 1. The majority of patients were adults ranging from 15 to 59 years, even if the +60 years group was less numerous in 2006. TB diagnosis was confirmed in most patients (69.1-71.7%) with smear positive TB being more frequent. HIV prevalence was similar (43%, 45% and 48% in 2005, 2006 and 2007, respectively) as well as the proportion of patients receiving Antiretroviral Therapy every year (78%, 75%, 79% in 2005, 2006 and 2007 respectively). Between 64-69.8% were HIV1, 20-23.5% were HIV2 and 10.2-11.7% HIV1/2.

**Table 1:** Patient's clinical characteristics

	2005		2006		2007		p-value
**Admissions **(N°)	427		346		549		

	***N°***	***%***	***N°***	***%***	***N°***	***%***	

**Gender**							
M	232	54.3%	203	58.8%	310	56.4%	0.480
F	195	45.7%	143	41.2%	239	43.6%	

**Age (years)**							
14-	53	12.4%	55	15.9%	92	16.8%	
15-59	325	76.1%	268	77.5%	395	71.9%	0.043
60+	49	11.5%	23	6.6%	62	11.3%	

**Symptoms on admission**							
Cough	392	92%	317	91.6%	499	90.8%	
Fever	366	85.7%	294	84.9%	486	88.5%	0.866
Chest pain	319	74.7%	252	72.8%	428	78%	
Weight loss	289	67.7%	248	71.6%	390	71%	

**Diagnosis**							
TB	306	71.7%	239	69.1%	390	71%	
*Pulmonary TB Smear+*	157		120		195		
*Pulmonary TB Smear-*	107		99		150		0.387
*Extra Pulmonary TB*	32		20		31		
Lung Disease No TB	94	22%	83	23.9%	110	20%	
Other	27	6.3%	24	7%	49	9%	

Mean Length of Stay (LOS) during the three years was 78 days (77, 87, 70 days in 2005, 2006, 2007 respectively).

### Outcomes

A significant reduction in mortality was observed from 2005 to 2007 (26.46% vs 18.76%, *p-value *0.003). Detailed outcomes for rainy season and dry season are presented in Table 2 and Table 3 respectively. The main determinant of the annual mortality reduction was shown to be the drop of rainy season mortality. In fact, the death/discharge ratio significantly decreased during the rainy season (0.79, 0.55 and 0.26 in 2005, 2006 and 2007 respectively; p-value 0.001) while remained fairly stable during the dry season (0.39, 0.37 and 0.32 in 2005, 2006 and 2007 respectively; p-value 0.647).

**Table 2:** Rainy season (July-October) outcomes of hospitalization in 2005, 2006 and 2007

Outcomes	2005	2006	2007	p-value
**Admissions**				
Total (n°)	158	130	173	
Mean per month	39,5	32,5	43,25	
	
**Deaths**				
Total (n°)	60	44	36	
Mean per month	15	11	9	
	
**Discharges**				
Total (n°)	76	80	137	
Mean per month	19	20	34.25	

**Mortality **(Death/Admission)%	37.9	33.8	20.8	0.0032
	(CI 95%:30.4%-45.54%)	(CI 95%:25.72%-41.98%)	(CI 95%:14.76%-26.86%)	

**Death/Discharge ratio**	0.78	0.55	0.26	0.001

**Table 3:** Dry season (November-June) outcomes of hospitalization in 2005, 2006, 2007

Outcomes	2005	2006	2007	p-value
**Admissions**				
Total (n°)	269	216	376	
Mean per month	33.62	27	47	
	
**Deaths**				
Total (n°)	53	51	67	
Mean per month	6.62	6,37	8,37	
	
**Discharges**				
Total (n°)	137	136	208	
Mean per month	17.12	17	26	

**Mortality **(Death/Admission)%	19.7	23.6	17.8	0.385
	(CI 95%: 14.95%-24.45%)	(CI 95%:17.95%-29.27%)	(CI 95%:13.95%-21.69%)	

**Death/Discharge ratio**	0.38	0.37	0.32	0.647

Deaths occurring during the first week of admission and therefore not influenced by malaria prophylaxis, were 41%, 28% and 47% during the rainy season in 2005, 2006 and 2007 respectively (p-value 0.178). Death/Discharge ratio calculated considering only deaths occurring after the first week, were again highly significant (p-value 0.001): 0.51, 0.40 and 0.14 in 2005, 2006 and 2007 respectively.

None of the patients abandoned treatment or asked to be discharged before completing the two months of TB intensive phase treatment or before clinical recovery.

Every health education class was attended by 96-99% of the admitted patients.

A reduction in Plasmodium Falciparum positive blood films was observed from the period August-November 2005 to August-November 2006 and 2007 (23.65% vs. 15.7% vs. 8.4% respectively, p-value 0.001) but the results were not registered on a regular basis.

### Costs

The overall cost of the entire malaria prevention program in 2007 was estimated to be €1050 (USD 1402). This figure includes: re-treatment of bed nets every 45 days along the year, outdoor and indoor disinfection performed during the rainy season and cotrimoxazole prophylaxis during the rainy season, with a mean cost of 2€/patient. Additional purchases were the spray pump and the facial mask that can be used for several years (€500 or USD 668). Bed nets were donated to the hospital by the Global Fund Malaria initiative over the three years.

## Discussion

Our study suggests that malaria prevention is feasible in African hospital settings and can involve both environment-focused and patient-focused interventions. Permethrin treated bed nets, outdoor and indoor environment disinfection and cotrimoxazole prophylaxis seem to have a cumulative protective effect on survival in severely ill TB patients.

Malaria and TB are a heavy burden to African health systems; the arrangement of integrated approaches of management with limited resources is still a challenge [1,5,6]. Seasonal variation in malaria related mortality [7,23] is well known in West-African countries and seasonal interventions have been effective in Gambia, Guinea-Bissau and Senegal [7,18,24]. Guinea-Bissau has one of the highest incidences of TB in the world [1] and malaria is an important death determinant in the country, since 45% of registered deaths are caused by malaria [25,26]. The majority of malaria related deaths occurs during the rainy season [25,26]. Therefore, it seems reasonable to target malaria prevention strategies to the rainy season.

All the three interventions seem to produce a certain benefit, even if environmental disinfection and cotrimoxazole prophylaxis seemed to be the most effective.

Permethrin treated bed nets reduce malaria infection offering both a mechanical and chemical barrier to mosquito bites and are efficacious in children and adults [16-18]. Insecticide treated bed nets and cotrimoxazole prophylaxis have shown to reduce malaria infection in HIV+ adults [19,27], children [28] and pregnant women [20,29,30]. Environmental disinfection, with indoor and outdoor spraying, is a useful tool to fight malaria in several countries [21,31,32]. A limit of vector control strategies has been the difficulty to deliver the reagents on time (i.e. at the beginning of the rainy season) to households spread in large areas and to perform re-treatment on schedule [31]. A hospital-based intervention overcomes these problems since patients are admitted in the same place for several months. Hospital outdoor and indoor spraying leads to a parasite intensity/density reduction and therefore can be an additional strategy to reduce the malaria burden in severely ill TB patients [20,32].

Cotrimoxazole prophylaxis administered to HIV+ patients has reduced malaria episodes in their HIV-seronegative household members [33] and was also efficacious in Mali with 99% prophylactic efficacy against malaria infection and disease in children [34]. Limited information are available on the role of cotrimoxazole for malaria prophylaxis in HIV-seronegative TB patients, even if once daily cotrimoxazole has reduced overall mortality in TB patients regardless of HIV status in South Africa [21] and also mortality and malaria infection in HIV+ TB patients in Abidjan [22].

The benefit of cotrimoxazole in our study may have been enhanced by the potential reduction of gastrointestinal illnesses, sepsis [34,35], pneumonia and toxoplasmosis [36,37], often leading causes of death in immunocompromised patients. These factors have not been directly investigated by our study and a prospective randomized study may address the specific correlations. Nevertheless, the poor clinical conditions of in-patients with severe or disseminated TB infection, usually malnourished, might justify the use of cotrimoxazole for short periods of time, regardless their HIV status.

Concerns regarding the induction of high resistance to cotrimoxazole in non-typhoid salmonella bacteria or in other opportunistic pathogens and the implications on the potential benefits of co-trimoxazole prophylaxis in HIV-infected individuals have been raised; however, the size of this phenomenon has not yet been proven to compromise the short and mid-term effectiveness of cotrimoxazole prophylaxis [38]. Therefore, there are no compelling reasons to believe that our approach could be of any harm from a public health perspective. The rapid evolution of antifolate resistance observed in malaria in sub-Saharan African countries -where bacterial resistance to cotrimoxazole is higher and cross-resistance between cotrimoxazole and sulfadoxine-pyrimethamine (SP) may impair SP efficacy for malaria treatment- raises questions about the durability of this strategy. A careful resistance analysis to evaluate the emergence of resistance to the drug should be performed to tailor malaria prevention.

The overall cost of vector control, personal protection and prophylaxis in our setting was really limited: only €2/patient allow a net gain in survival with a reduction of death/discharge ratio from 0.76 to 0.26. Surely, there is a higher cost of malaria in-patient care compared to prevention [5]. Malaria's economic impact is enormous [6] and the high direct cost for treating illness justifies efforts to improve coverage of preventive measures. Malaria preventive strategies targeting high risk groups -as severely ill TB patients- in high burden seasons -as the rainy season- could reduce diagnostic and treatment direct cost for families (where user fees are applied) and health services (where health care is provided without charge) while reducing mortality.

Population's clinical characteristics, TB pattern and HIV prevalence were fairly constant along the three years of the intervention. Mortality during the first week of admission, and therefore not influenced by malaria prophylaxis, was also always above 28% suggesting that late presentation to the health center was a major factor for in-hospital death. Unfortunately, it is a common problem in TB facilities across Africa [39,40]. There was no effect on mortality by age in our group of patients, even if susceptibility to severe malaria is affected by age, but this could be due to the relative small proportion of children in our group.

No drop-outs were observed. This is a remarkable data considering the high drop-out rates in many TB programs in Africa [1]. The free access to diagnosis and to all components of treatment (pharmacological and nutritional) that was offered by the hospital in those years was surely a major determinant for good compliance. Repeated health education (once a week) performed by trained personnel was also attractive for patients (96-99% of admitted patients attended each class) and reinforced sleeping under bed nets as well other healthy behaviors.

Our study has several limits. Firstly, it is an observational study and therefore it doesn't have the power of a randomized study. Nevertheless, even if it doesn't compare the three different strategies in a randomized fashion, the three-step intervention testes them subsequently and suggests the additive benefit of each successive one (death/discharge ratio dropped from 0.79 to 0.55 to 0.26). Secondly, malaria was not evaluated as a secondary outcome and therefore there is no direct demonstration that the reduction in mortality was due to the reduction in the number of both malaria episodes and malaria related mortality. Even so, all treatment protocols and main clinical characteristic of patient population remained fairly unchanged along the three years, and the only modifying interventions were related to malaria prevention. A reduction in positive blood films was indeed observed from the period August-November 2005 to August-November 2006 and 2007 (23.65% vs. 15.7% vs. 8.4% respectively, p-value 0.001), and even if the results were not registered on a regular basis and therefore are not completely reliable, they contribute to the hypothesis that malaria reduction was a determinant of reduced mortality. It is unlikely that patients died partly as a result of ineffective treatment for their malaria because chloroquine resistance is scarce in the country probably due to the higher chloroquine dosages routinely used [41].

## Conclusion

In conclusion, our prospective observational study suggests that malaria vector control, personal protection and cotrimoxazole prophylaxis can be a safe and low cost measure to reduce in-hospital mortality in TB patients admitted in reference centers due to their poor clinical status and/or disseminated disease. Free care and health education have a role in reducing drop-outs in TB and malaria hospital-based programs. Prospective randomized trials are needed to confirm our findings and understand the role of cotrimoxazole in reducing overall morality and malaria related mortality in TB patients.

## Competing interests

The authors declare that they have no competing interests.

## Authors' contributions

CR: designed the study, interpreted the data and wrote the manuscript; PM: interpreted the data and reviewed the manuscript; BF: designed the study and collected the data; VCS: performed the study and collected the data; ALA: performed the study and collected the data; VF: performed the study and collected the data; TS: performed statistical analysis; GC: reviewed the manuscript; RF: designed the study, interpreted the data and reviewed the manuscript.

All authors read and approved the final manuscript

## Pre-publication history

The pre-publication history for this paper can be accessed here:

http://www.biomedcentral.com/1471-2334/11/57/prepub
